# Miliary Histoplasmosis in a Renal Transplant Patient

**DOI:** 10.7759/cureus.19338

**Published:** 2021-11-07

**Authors:** Jorge Verdecia, Ashlan J Kunz Coyne, Shaorinkumar Patel, Melissa Oye, Malleswari Ravi, Michael Sands

**Affiliations:** 1 Infectious Disease, University of Florida College of Medicine – Jacksonville, Jacksonville, USA; 2 Pharmacology, University of Florida College of Medicine – Jacksonville, Jacksonville, USA; 3 Internal Medicine, University of Florida College of Medicine – Jacksonville, Jacksonville, USA; 4 Infectious Diseases, University of Florida College of Medicine – Jacksonville, Jacksonville, USA

**Keywords:** non-endemic histoplasma, renal transplant, acute miliary histoplasmosis, solid organ transplant, histoplasmosis

## Abstract

Solid organ transplant (SOT) recipients are at increased risk of opportunistic infections due to significant T-cell immune dysfunction. The incidence of clinical disseminated histoplasmosis is rare, and its variable clinical presentation and response to therapy make it challenging to treat with resultant high mortality. A high index of clinical suspicion is necessary, especially in non-endemic areas. We report our clinical experience treating a 63-year-old renal transplant patient on immunosuppressive therapy with late-onset acute miliary histoplasmosis initiated on liposomal amphotericin B (L-AmB).

## Introduction

Clinical disseminated histoplasmosis in solid organ transplant (SOT) recipients is rare, with an estimated incidence of <0.5% even in endemic areas [1,2]. Disseminated disease with pulmonary involvement is the most common, with the highest risk period being the first year after transplant, although cases have been reported up to 20 years after transplantation [2,3]. The estimated mortality of disseminated histoplasmosis is 10%, with most deaths occurring within one month of diagnosis [2]. Histoplasma urine antigen is the most sensitive test for diagnosis, with higher sensitivity for disseminated disease than pulmonary disease alone [4]. We present a case of a renal transplant patient diagnosed with late-onset acute miliary histoplasmosis started on liposomal amphotericin B (L-AmB) but died 10 days later secondary to gastrointestinal bleeding.

## Case presentation

**Figure 1:**
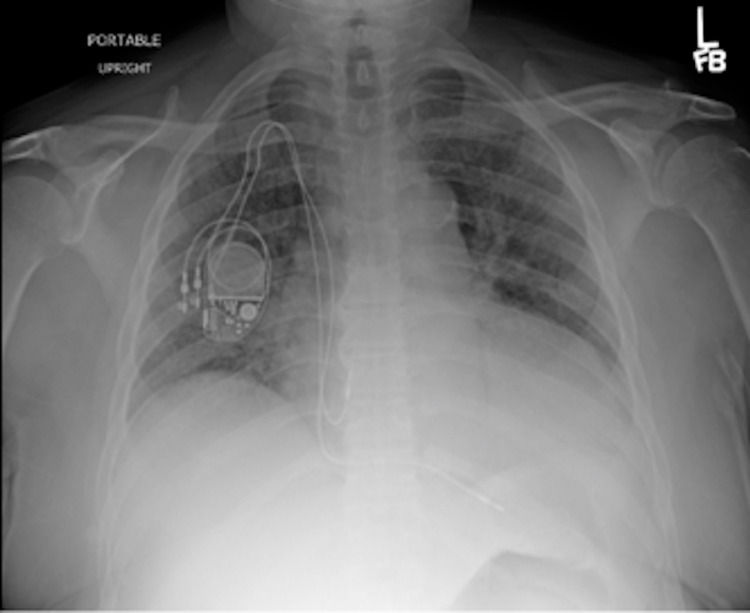
Stable cardiomegaly with mild pulmonary vascular congestion and diffuse interstitial edema or possibly pneumonia

**Figure 2:**
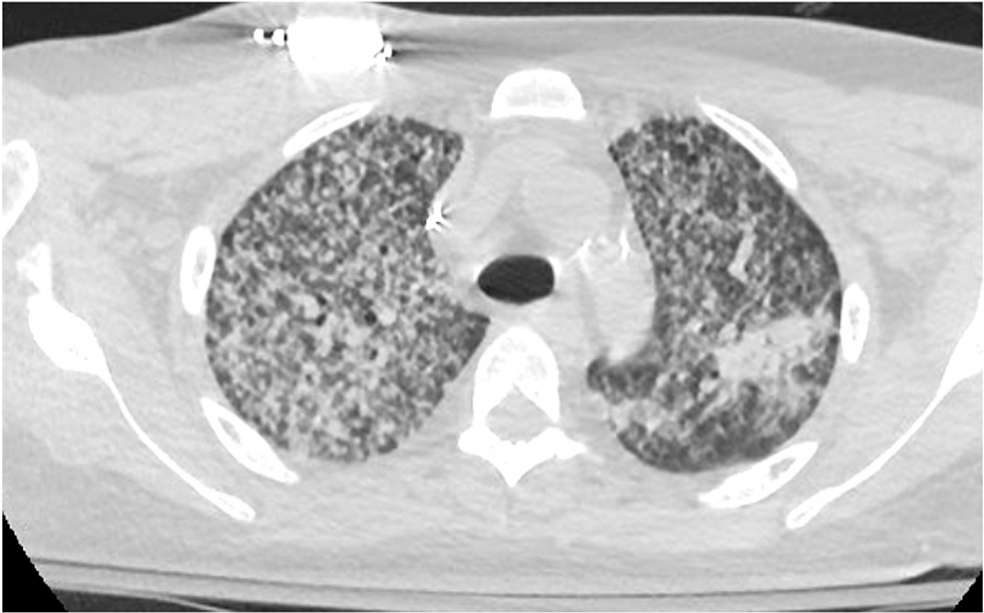
Innumerable bilateral reticulonodular opacities throughout both lungs with areas of consolidation

**Figure 3:**
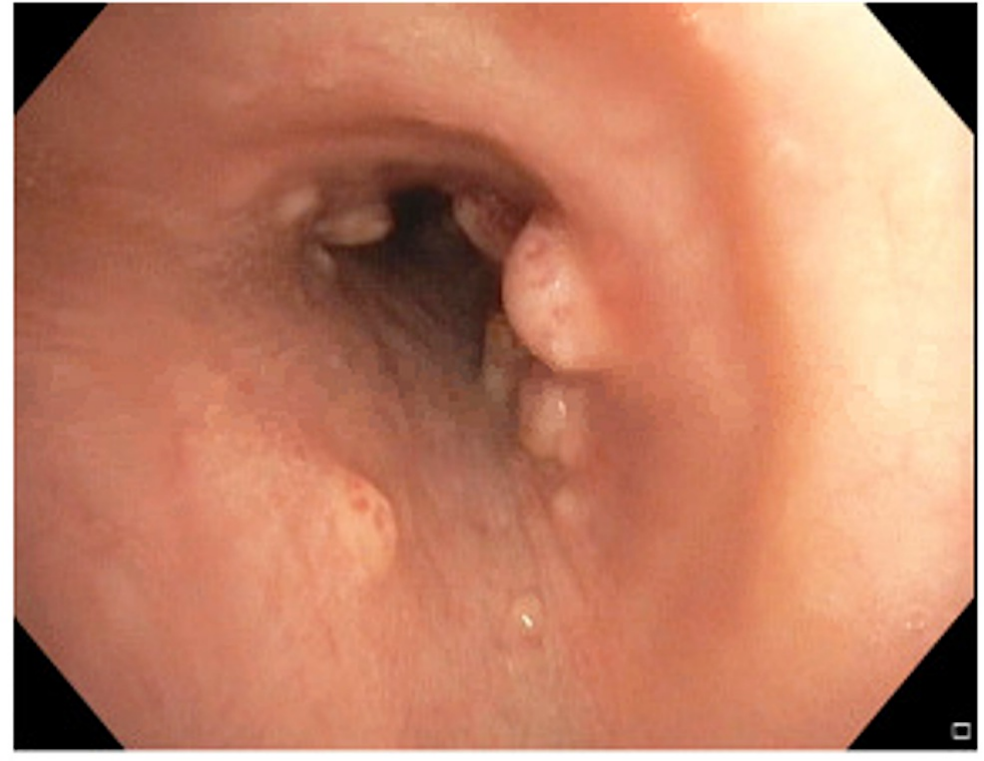
Diffuse endobronchial nodules within the right main bronchus

A 63-year-old male presented with a dry cough, weakness, and fatigue of three-week duration. The patient’s prior medical history was significant for type 2 diabetes mellitus requiring insulin for the last 10 years; renal transplant in 2009 on tacrolimus 0.5 mg twice daily, mycophenolate 360 mg twice daily, and prednisone 20 mg daily; and chronic allograft nephropathy identified via transplant biopsy in 2013. His wife was from Haiti, but the patient denied any international travel in the preceding 10 years. An initial examination showed a temperature of 100.7°F, pedal edema, mild confusion, serum creatinine at his baseline of 3.5 mg/dL, and a supratherapeutic tacrolimus level of 4.7 ng/mL. His chest X-ray demonstrated likely pulmonary edema or possibly pneumonia (Figure 1). Intravenous (IV) vancomycin and cefepime were started for the empiric treatment of bacterial pneumonia, but after 48 hours of IV antibiotics, he remained persistently febrile and required supplemental oxygen. His renal graft function deteriorated, and hemodialysis was initiated. On hospital day 5, a chest CT showed innumerable bilateral reticulonodular opacities throughout both lungs with areas of consolidation, greatest at the upper lung zones (Figure 2). On bronchoscopy, he had numerous endobronchial nodules (Figure 3), and subsequent bronchoalveolar lavage cultures grew *Enterococcus* spp. and yeast, which was later identified as *Histoplasma capsulatum*. Tissue pathology of the endobronchial lesions on Grocott's methenamine silver (GMS) staining demonstrated intracellular yeast, and his urine histoplasma antigen was positive; hence, L-AmB was initiated. A spinal fluid examination collected after four days of therapy showed CSF with no white blood cells, elevated protein at 79 mg/dL (normal range: 15-45 mg/dL), and glucose at 58 mg/dL (normal range: 60-80 mg/dL), with serum glucose at 215 mg/dL. The histoplasma antigen was not done in the spinal fluid. A head CT with IV contrast showed stable chronic lacunar infarcts within the bilateral basal ganglia and right pons but with no acute intracranial hemorrhage, mass effect, or territorial infarct and no abnormal intracranial enhancement. A brain MRI was not performed given the improvement in mentation. Fungal blood cultures remained negative. After 10 days of L-AmB therapy, the patient’s mentation returned to normal, but unfortunately, the patient expired from a gastrointestinal hemorrhage. The family declined an autopsy, and the cause of the bleeding remains unclear.

## Discussion

*Histoplasma capsulatum *is a soil-dwelling thermally dimorphic fungus that grows as a mold (macro- and microconidia) in nature and in culture at room temperature but converts into a small (1-5 μm in diameter) yeast cell at 37°C and during the invasion of the host cells [1].

It has a worldwide distribution (except Antarctica) [5], and in the United States (US), it is endemic to the areas of the Ohio-Mississippi River valleys based on information provided in the 1950s using skin test reactivity [5]. Since then, cases have been reported in areas with low endemicity, including Montana, Nebraska, the Northern Great Lakes area, Minnesota, Florida, and South Carolina [3,6]. This changing epidemiology could be due to various factors such as soil disruption by excavation or construction, the HIV/AIDS epidemic (AIDS-defining illness), or the use of immunosuppressive medication [4,6]. Despite being described as the most common endemic mycosis in the US and immunosuppressed patients being at increased risk of opportunistic infections due to impaired cell-mediated immunity (CMI), histoplasmosis is rare in SOT recipients [1,2]. Even so, histoplasmosis has been described among all types of SOT recipients, although differences in the attack rate and disease incidence were noted between groups, with lung and heart transplant recipients having higher incidence [1,4]. The first year after transplant is the period of most significant risk, likely due to the high level of immunosuppression, but cases have been reported up to 20 years after transplantation [2].

Histoplasmosis in transplant recipients is acquired through primary infection, usually via inhalation of microconidia through a pulmonary route. Previous infections can reactivate in the recipient posttransplantation, or a donor-derived infection can occur [4]. Microconidia convert to a yeast form in pulmonary macrophages and disseminate widely. A normal host usually remains asymptomatic or may suffer influenza-like illness. Still, immunocompromised hosts frequently become symptomatic, resulting in various clinical manifestations, the most common form being progressive disseminated histoplasmosis (PDH) [1]. The clinical features of PDH in SOT recipients are nonspecific. They may include subacute febrile illness, shortness of breath, and fatigue. Pulmonary involvement is seen in the majority of patients as diffuse reticulonodular or miliary infiltrates. Extrapulmonary sites include the liver, spleen, bone marrow, central nervous system (CNS), and gastrointestinal tract [3]. The illness most commonly presents in an occult manner in the transplant population, with the burden of disease often out of proportion to the severity of symptoms at initial presentation [4].

A complex presentation of histoplasmosis is the involvement of the central nervous system (CNS), most commonly seen in disseminated histoplasmosis infection with an incidence rate of 5%-10% [7]. CNS manifestations can include meningitis involving the basilar meninges, acute meningitis, encephalitis, small ring-enhancing lesions, abscess, and stroke due to infected emboli [8]. Brain imaging is abnormal in most cases, with MRI being more sensitive than CT scan, although, in one study, greater than 20% of patients had no significant findings on head imaging [7]. The most common CSF finding is lymphocytosis, but about a third of patients have normal white cell counts, and the most sensitive method for diagnosis is an antibody or antigen detection in the CNS [7]. Morbidity and mortality are extremely high due to relapsing CNS infection, with one group study showing a case fatality rate of 39% [7].

Of the currently available diagnostic tests, none have 100% accuracy. Growth of organisms from clinical specimens is the definitive test, but culture may take up to four weeks. Visualizing morphologically consistent yeast forms in tissue specimens is a faster diagnosis, but organism misidentification remains possible [3]. Additionally, obtaining samples may not be possible in a clinically unstable patient. Hence, urine histoplasma antigen remains a clinically practical test with a sensitivity that approaches 90% and increases in immunocompromised hosts due to a higher burden of infection. However, specificity can be less in immunocompromised patients due to cross-reactivity with other pathogens and antithymocyte globulin [3,4]. Histoplasma antibody testing is of limited utility in SOT due to impaired antibody responses, at 30% [4,7].

The recommended treatment of acute and chronic disseminated histoplasmosis according to the 2007 Infectious Diseases Society of American (IDSA) guidelines consist of a lipid formulation of liposomal amphotericin B (L-AmB) (3-5 mg/kg daily intravenously for one to two weeks), followed by oral itraconazole (200 mg thrice daily for three days and then 200 mg twice daily, for a total of 12 weeks) [9]. Treatment of histoplasmosis with L-AmB comes with nephrotoxicity concerns and possible allograft loss, while alternative therapy puts the patient at risk for suboptimal treatment [10,11]. Additional pharmacokinetic (PK) and drug-drug interaction considerations must be assessed in kidney transplant patients upon initiation and throughout therapy. Some factors associated with L-AmB use and subsequent acute kidney injury (AKI) include concomitant administration of renin-angiotensin system blockers, catecholamines or immunosuppressants, L-AmB doses ≥ 3.5 mg/kg/day, and serum potassium < 3.5 mEq/L immediately before L-AmB administration [12]. In patients taking concomitant itraconazole and tacrolimus, dose adjustment and therapeutic drug monitoring (TDM) considerations must be made. Itraconazole inhibits CYP3A4 and P-glycoprotein, resulting in increased tacrolimus concentrations, which increases the potential for adverse effects because of excessive immunosuppression and toxicity (e.g., nephrotoxicity and neurotoxicity) [13,14]. Tacrolimus trough concentrations should be assessed upon the initiation of itraconazole and subsequent dose adjustments to ensure that concentrations are within the narrow therapeutic window [15,16]. Notably, itraconazole oral formulations (e.g., solution, capsule, and tablet) have inherent PK differences and should not be used interchangeably [13].

Based on the poor predictive value of a positive serology and low likelihood of subsequent infection, pre‐transplant serologic and/or radiologic screening for prior histoplasmosis infection in endemic areas is not recommended, in contrast to coccidioidomycosis [4]. The primary prophylaxis for histoplasmosis in the posttransplant setting is not recommended [4]. Individuals who have recovered from active histoplasmosis during the two years before the initiation of immunosuppression may be considered for secondary azole prophylaxis, typically with oral itraconazole 200 mg daily [4].

## Conclusions

Unlike immunocompetent hosts, SOT recipients with posttransplant histoplasmosis (PTH) often present with disseminated disease and have attributable mortality of approximately 10%. A combination of various tests (culture, antigen tests, nucleic amplification tests, etc.) should be used to optimize diagnostic yield. A high index of suspicion must be present in non-endemic areas for the diagnosis to be made, as many presenting symptoms can mimic other common diagnoses.
